# Supplementary material for: Vaccination History, Body Mass Index, Age, and Baseline Gene Expression Predict Influenza Vaccination Outcomes
Source: Viruses. 2022 Nov 4;14(11):2446. doi: 10.3390/v14112446 (PMC9697051; doi:10.3390/v14112446)
Supplement: Supplementary file 1 [file viruses-14-02446-s001.zip › viruses-1917612-supplementary figures.pdf]

viruses

MDPI

Supplementary Figures

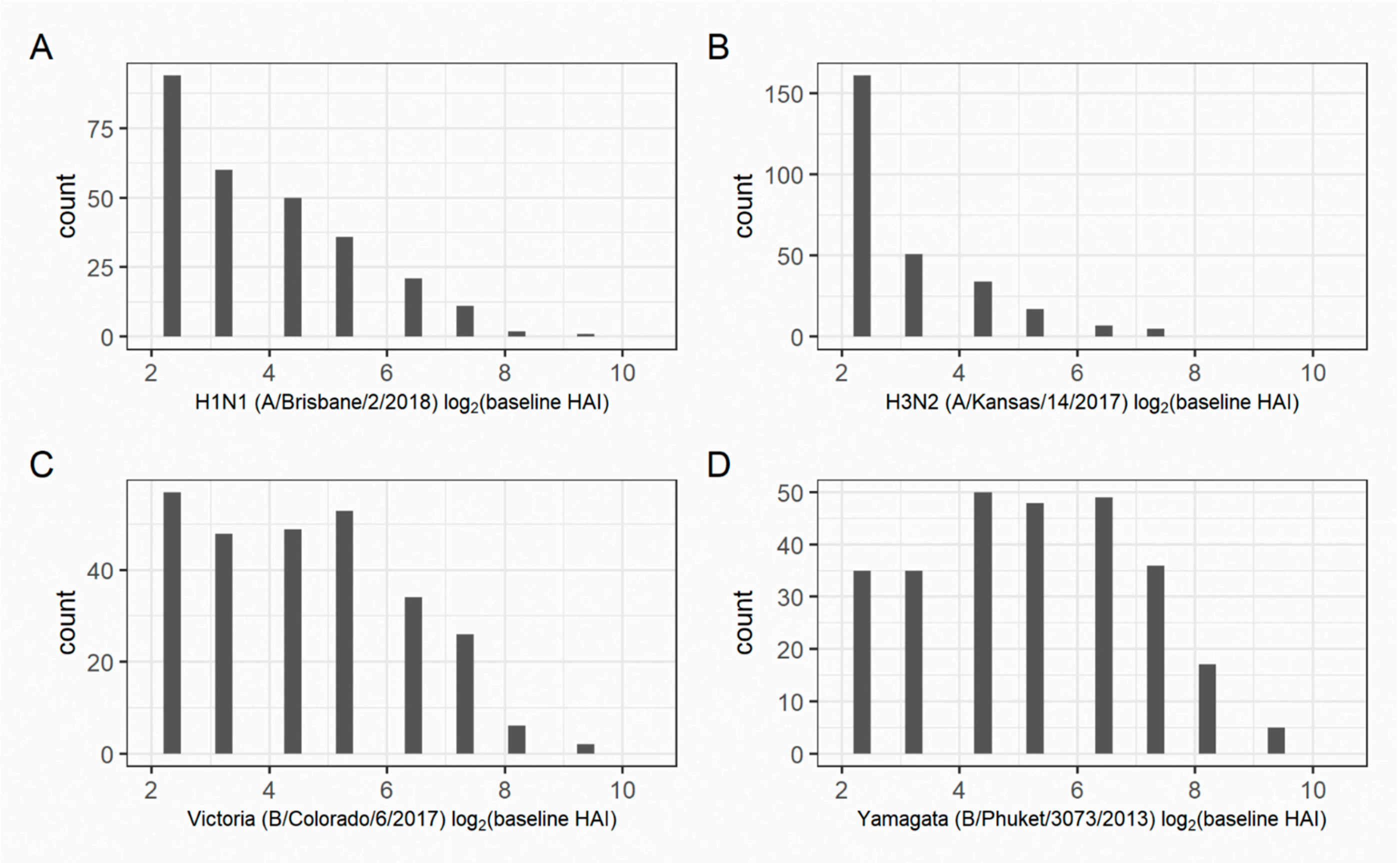

Figure S1. Distribution of baseline HAI for each influenza strain among study participants. The number of individuals (count) with the corresponding HAI value prior to vaccination for the **A**) H1N1, **B**), H3N2, **C**) Victoria, and **D**) Yamagata strains included in the vaccine formulations. Values for all 275 individuals are reported for each of the four strains.

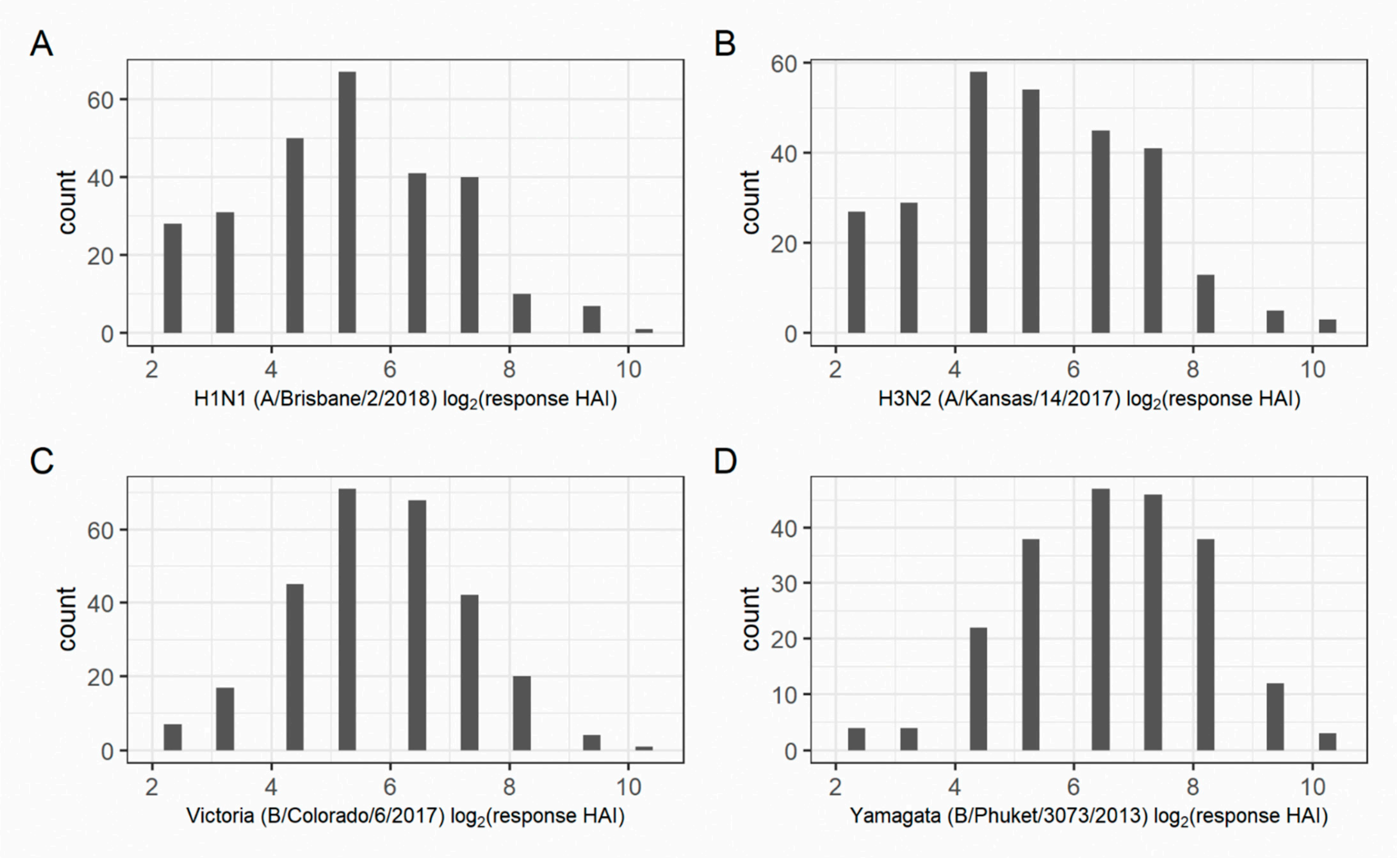

Figure S2. Distribution of response HAI for each influenza strain among study participants**.** The number of individuals (count) with the corresponding HAI value 28 days after vaccination for the **A**) H1N1, **B**), H3N2, **C**) Victoria, and **D**) Yamagata strains included in the vaccine formulations. Values for all 275 individuals are reported for each of the three of the strains, but only the 214 individuals who received the standard dose formulation containing the Yamagata component are shown in panel D.

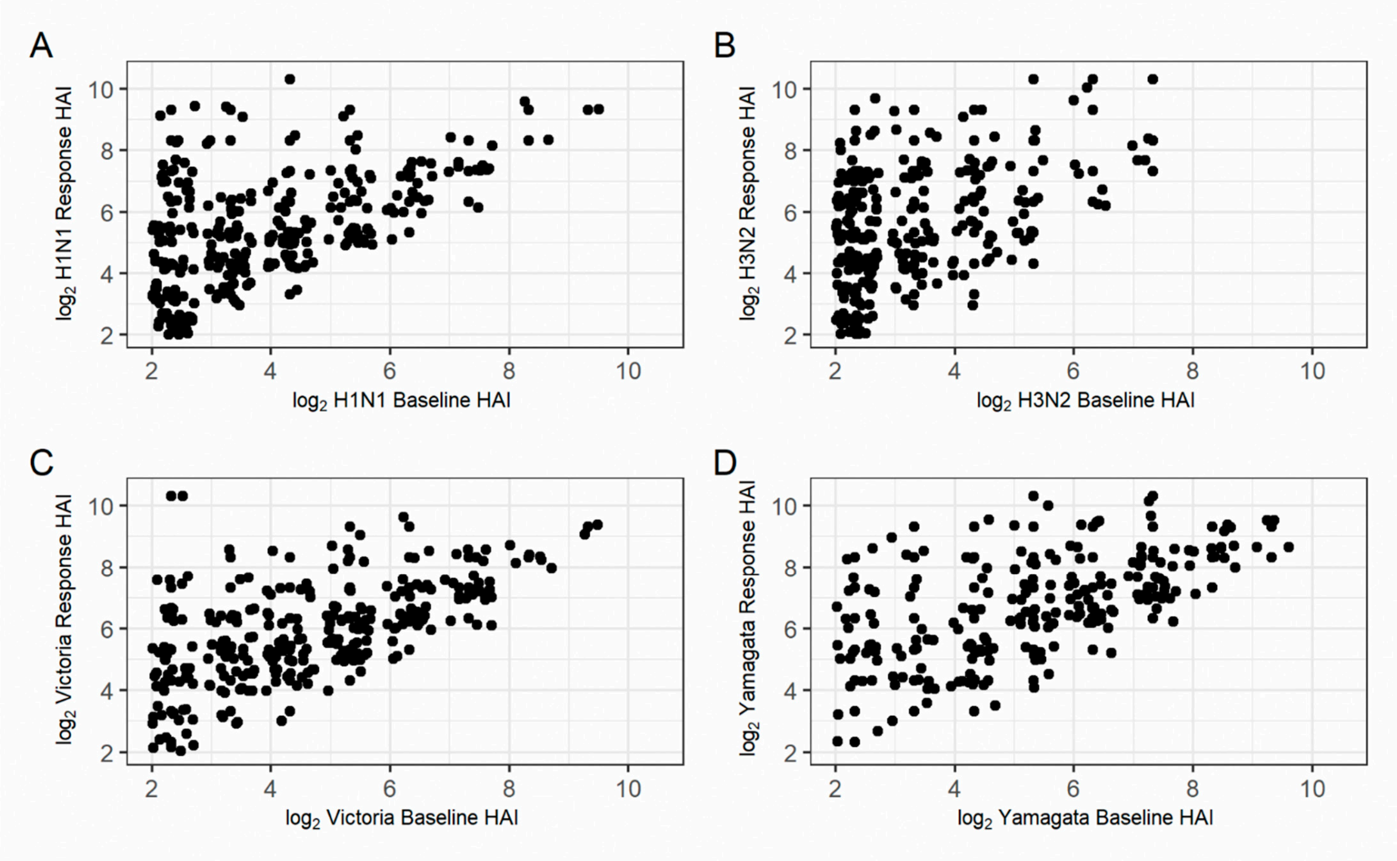

Figure S3. Relationship between baseline HAI and response HAI for each strain**.** The baseline HAI, measured prior to vaccination and the corresponding HAI value 28 days after vaccination for the **A**) H1N1, **B**), H3N2, **C**) Victoria, and **D**) Yamagata strains included in the vaccine formulations. Values for all 275 individuals are reported for each of the three of the strains, but only the 214 individuals who received the standard dose formulation containing the Yamagata component are shown in panel D. A small amount of random noise was added to each value for visualization purposes to avoid overplotting points.

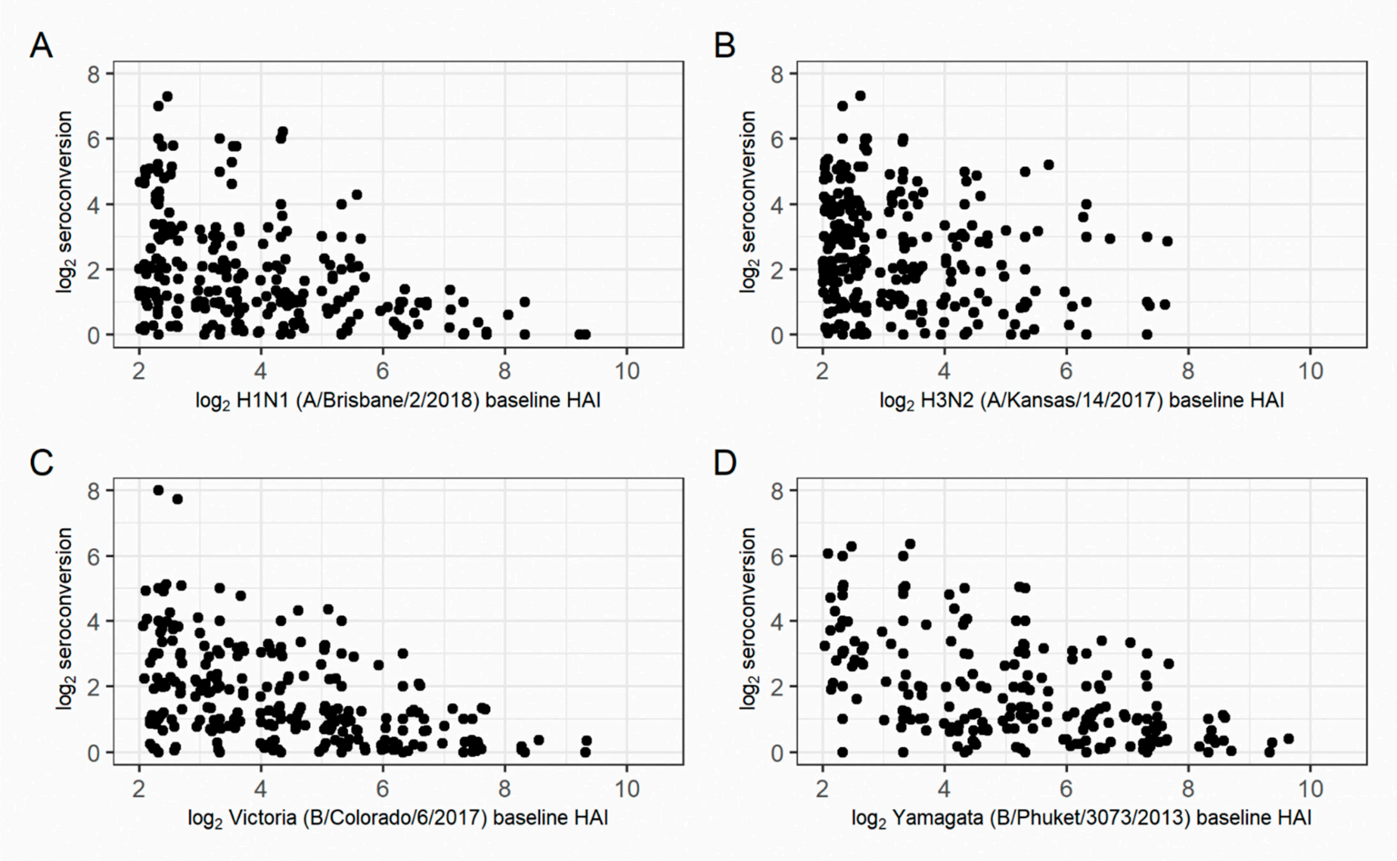

Figure S4. Relationship between baseline HAI and seroconversion score for each strain. The baseline HAI, measured prior to vaccination and the corresponding seroconversion score for the **A**) H1N1, **B**), H3N2, **C**) Victoria, and **D**) Yamagata strains included in the vaccine formulations. Values for all 275 individuals are reported for each of the three of the strains, but only the 214 individuals who received the standard dose formulation containing the Yamagata component are shown in panel D. A small amount of random noise was added to each value for visualization purposes to avoid overplotting points.

.

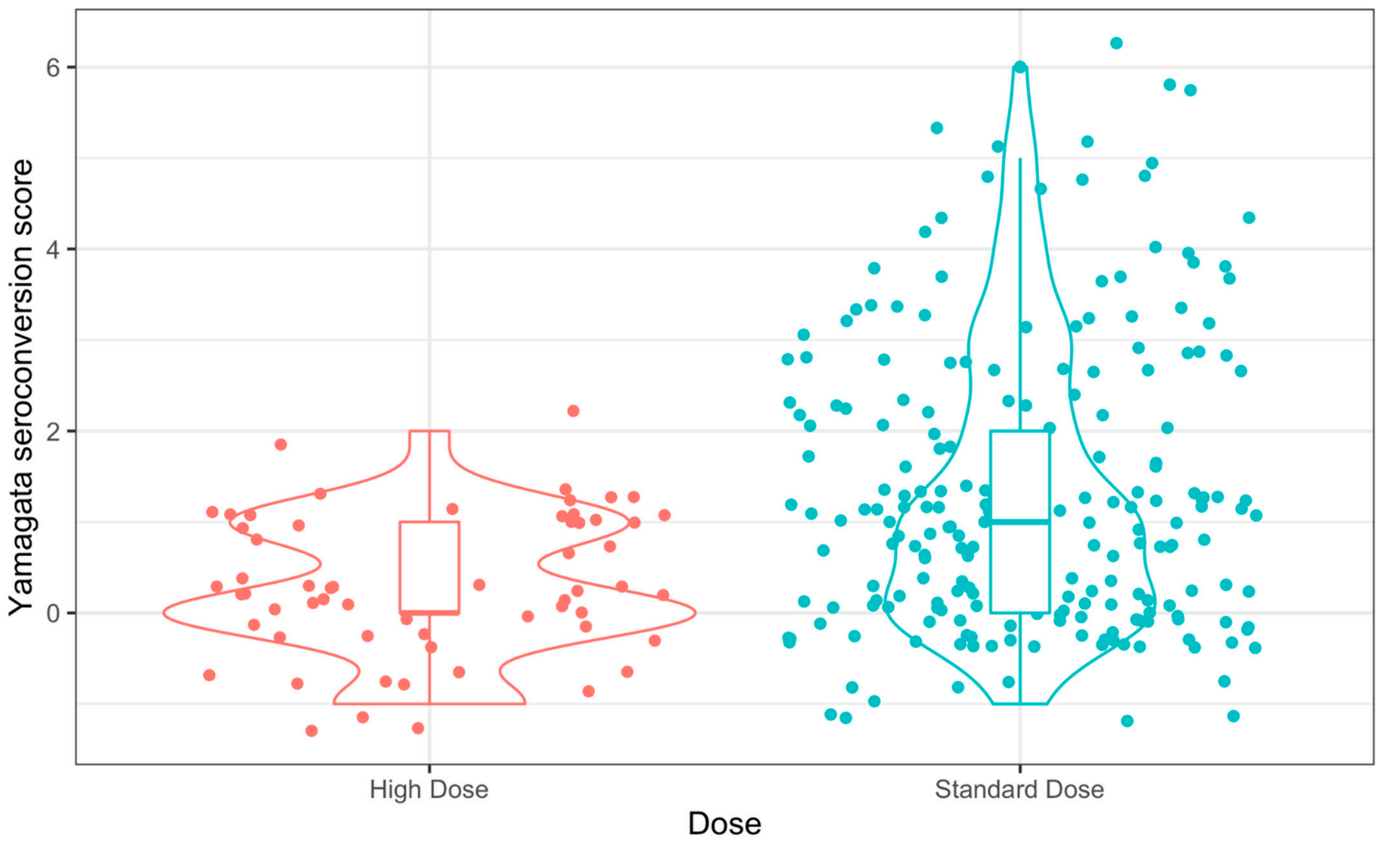

Figure S5. Seroconversion differences between vaccine formulations**.** The distribution of seroconversion scores for the Yamagata strain for those individuals (n = 61) who received the high dose formulation in which the Yamagata strain was excluded and those individuals (n = 214) who received the low dose formulation in which the Yamagata strain was included. A small amount of random noise was added to each value for visualization purposes to avoid overplotting points.

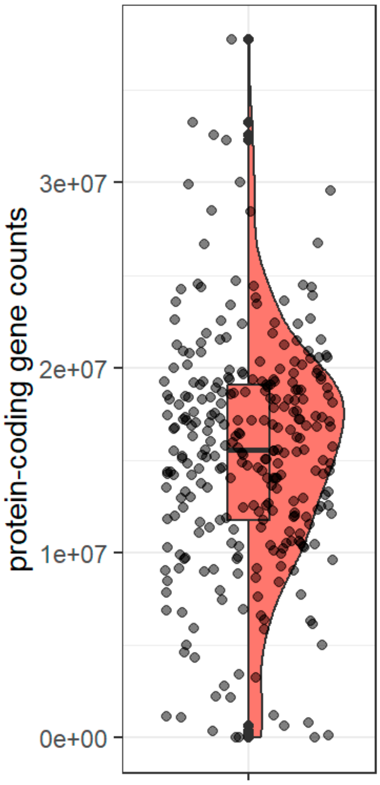

Figure S6. RNAseq read count distribution across 275 study participants**.** A median of 15 million sequence reads per individual mapped to protein coding genes.

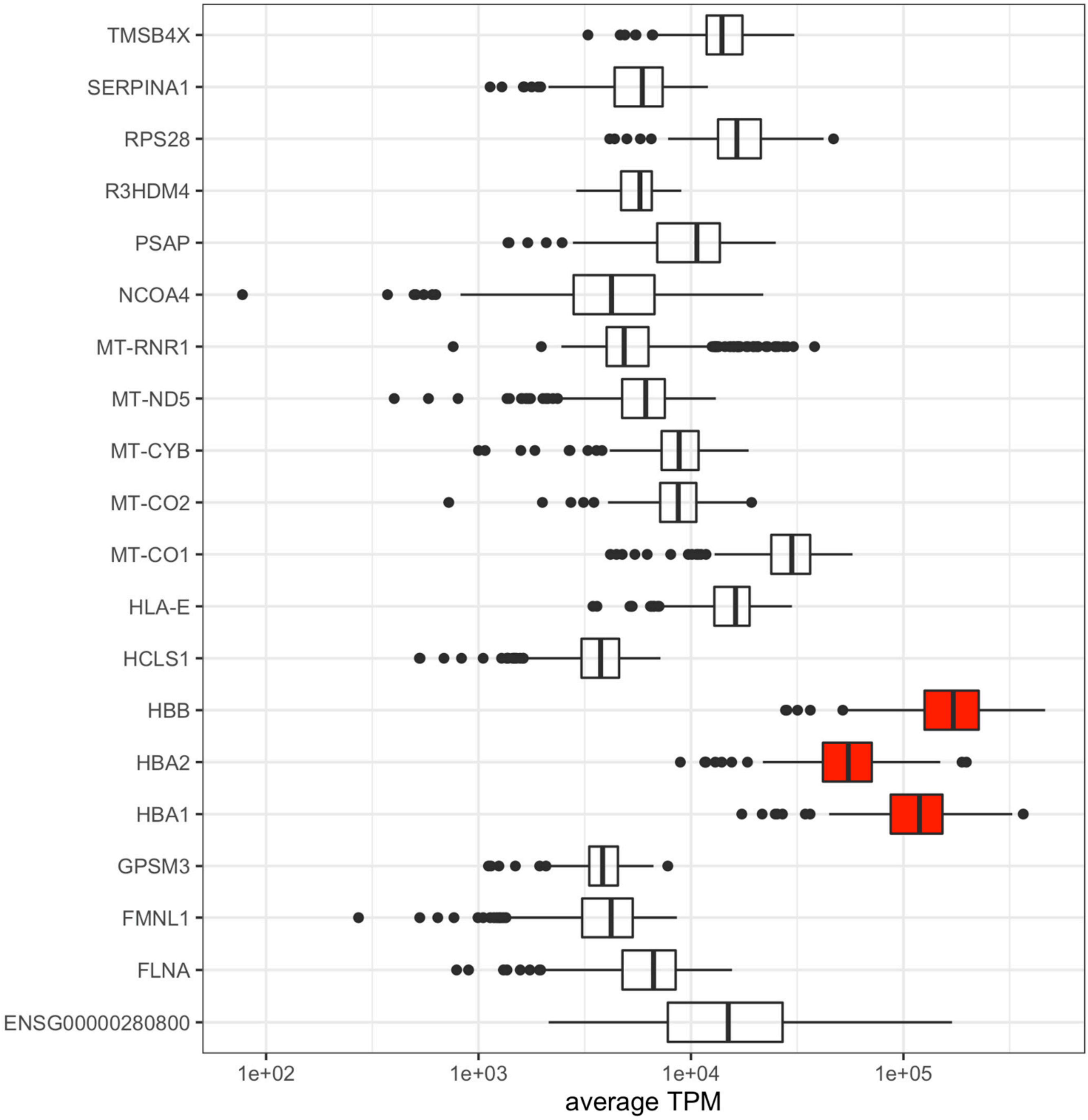

Figure S7. Genes with highest average transcripts per million (TPM) across all study participants. The aggregate counts of three genes, encoding hemoglobin, account for close to 50% of total transcripts reads.

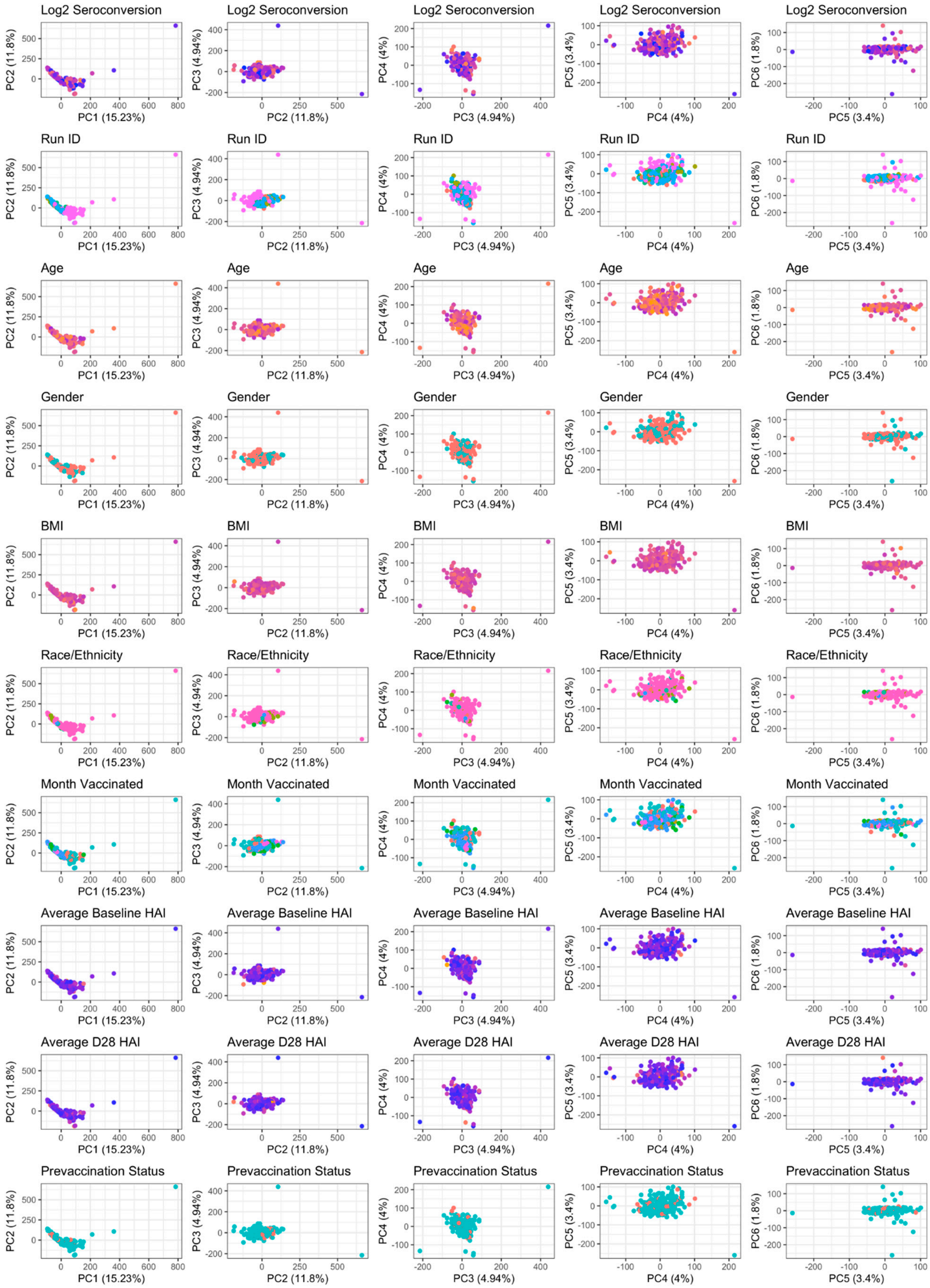

Figure S8. Principal component analysis of per sample transcript counts from RNA sequencing data.

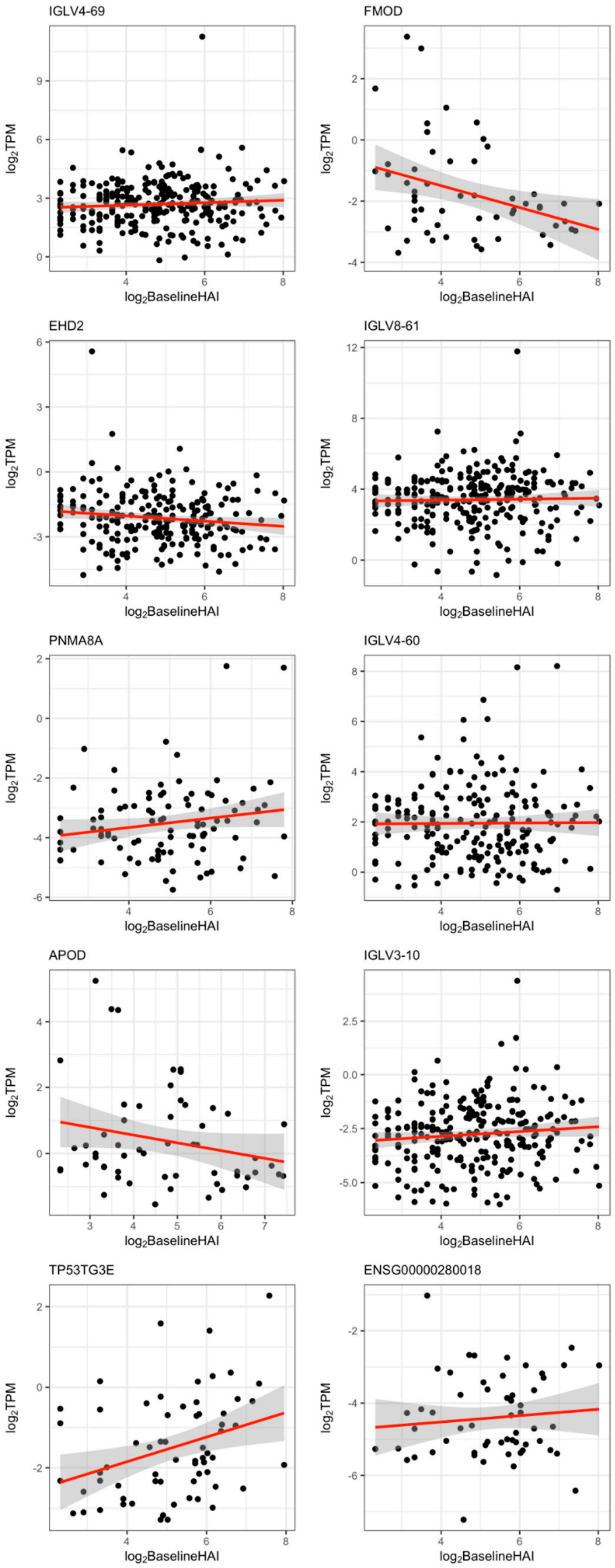

Figure S9. Top 10 DE genes as a function of baseline HAI ranked by FDR. Genes were selected for visualization on the basis of statistical significance and detection in at least 50 participants.

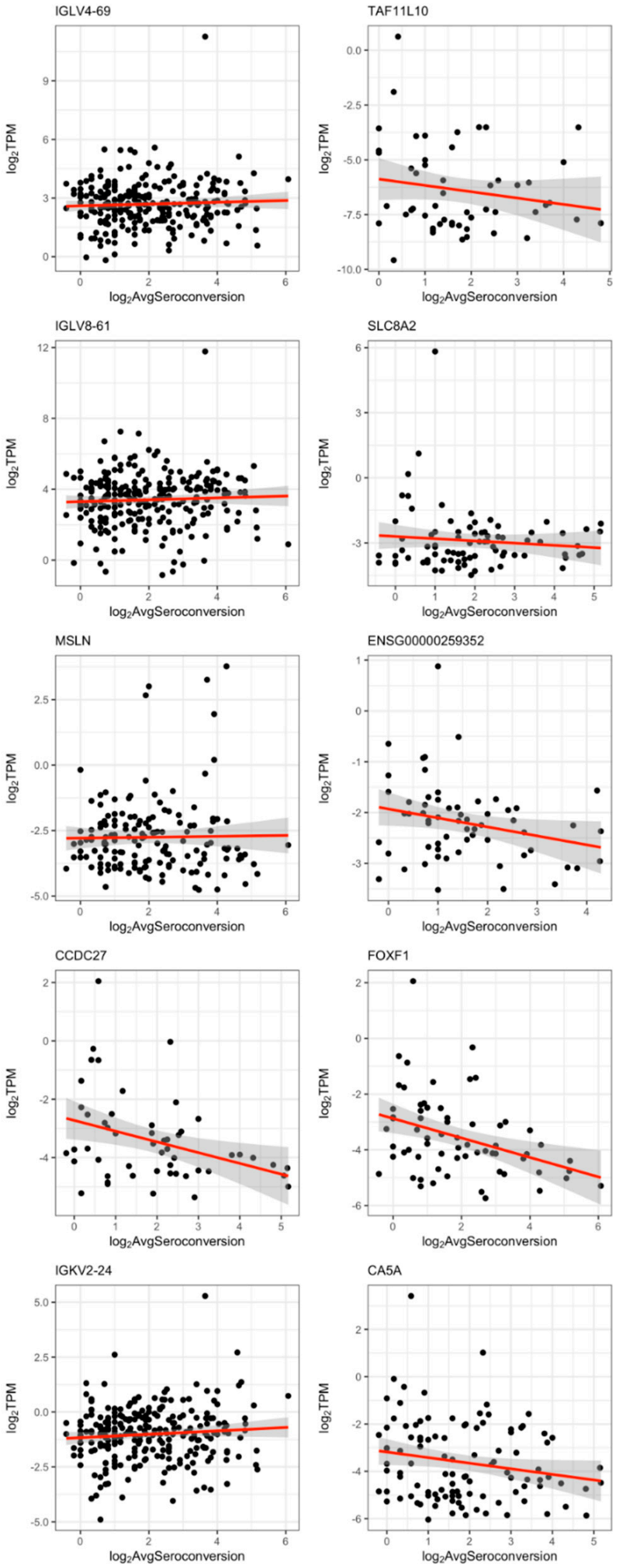

Figure S10. Top 10 DE genes as a function of seroconversion ranked by FDR. Genes were selected for visualization on the basis of statistical significance and detection in at least 50 participants.

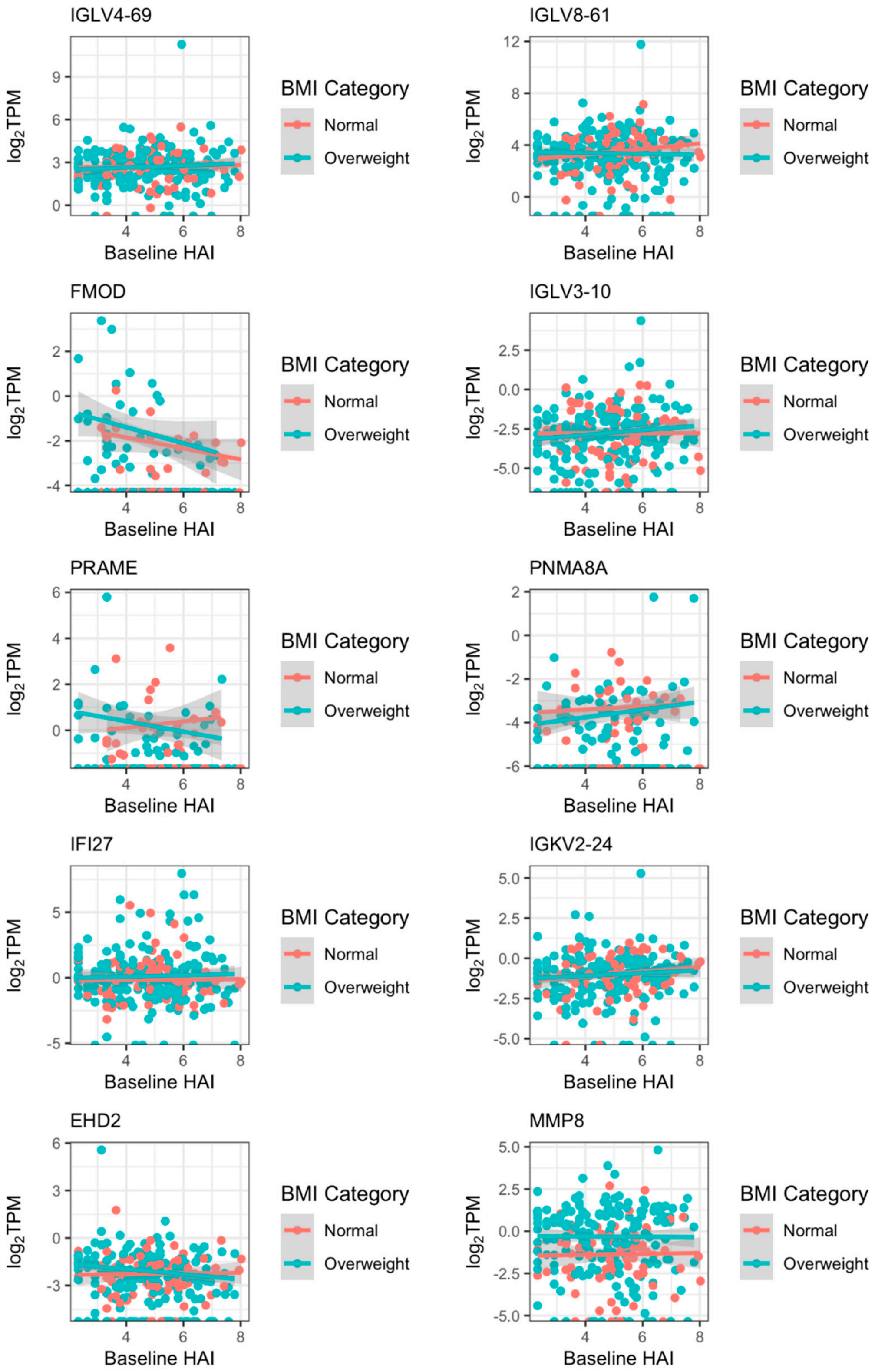

Figure S11. Top 10 DE genes as ranked by correlation coefficient to the interaction variable of initial HAI and BMI. Genes were selected for visualization on the basis of statistical significance and detection in at least 50 participants.

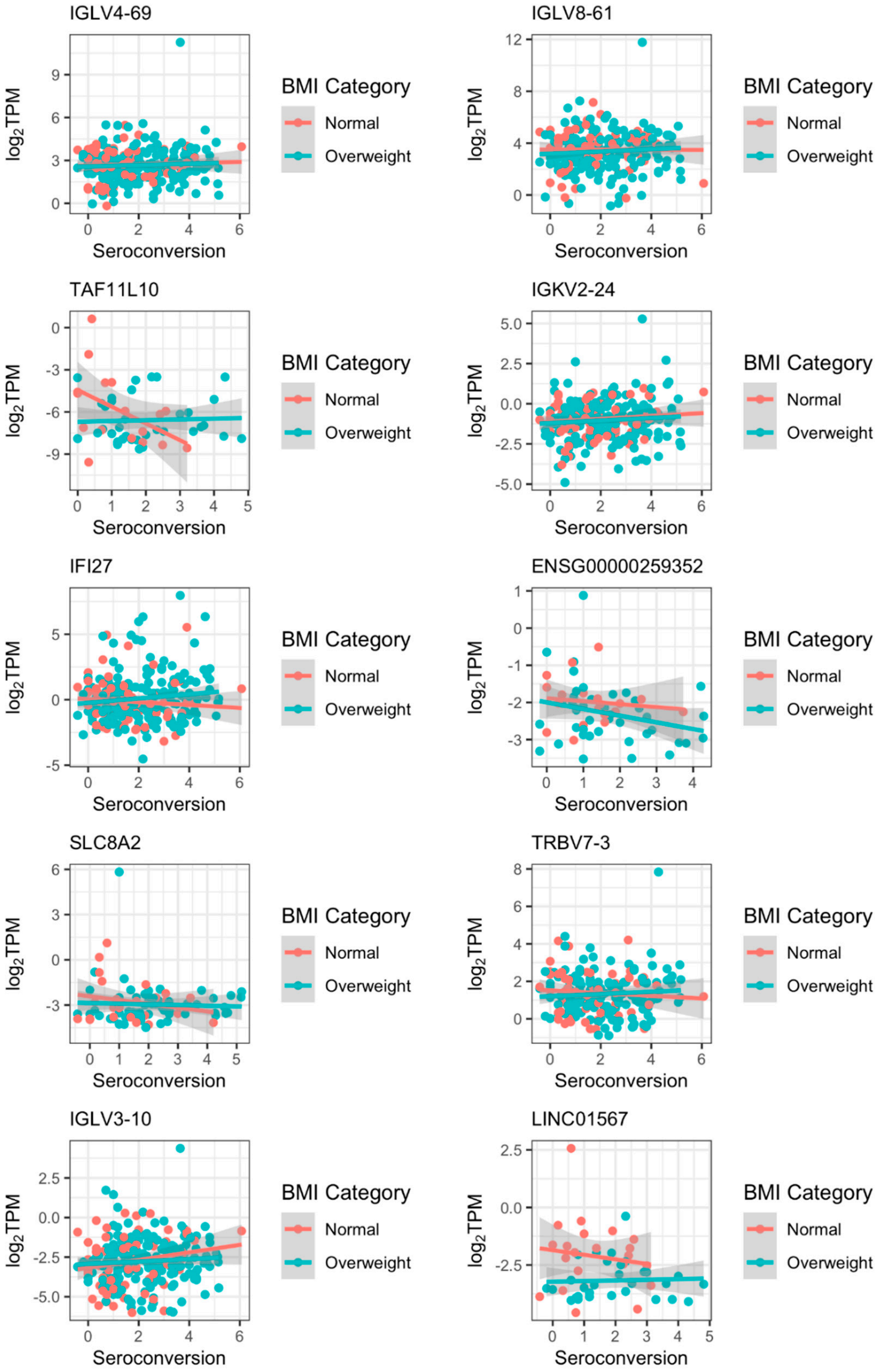

Figure S12. Top 10 DE genes as ranked by correlation coefficient to the interaction variable of seroconversion score and BMI. Genes were selected for visualization on the basis of statistical significance and detection in at least 50 participants.

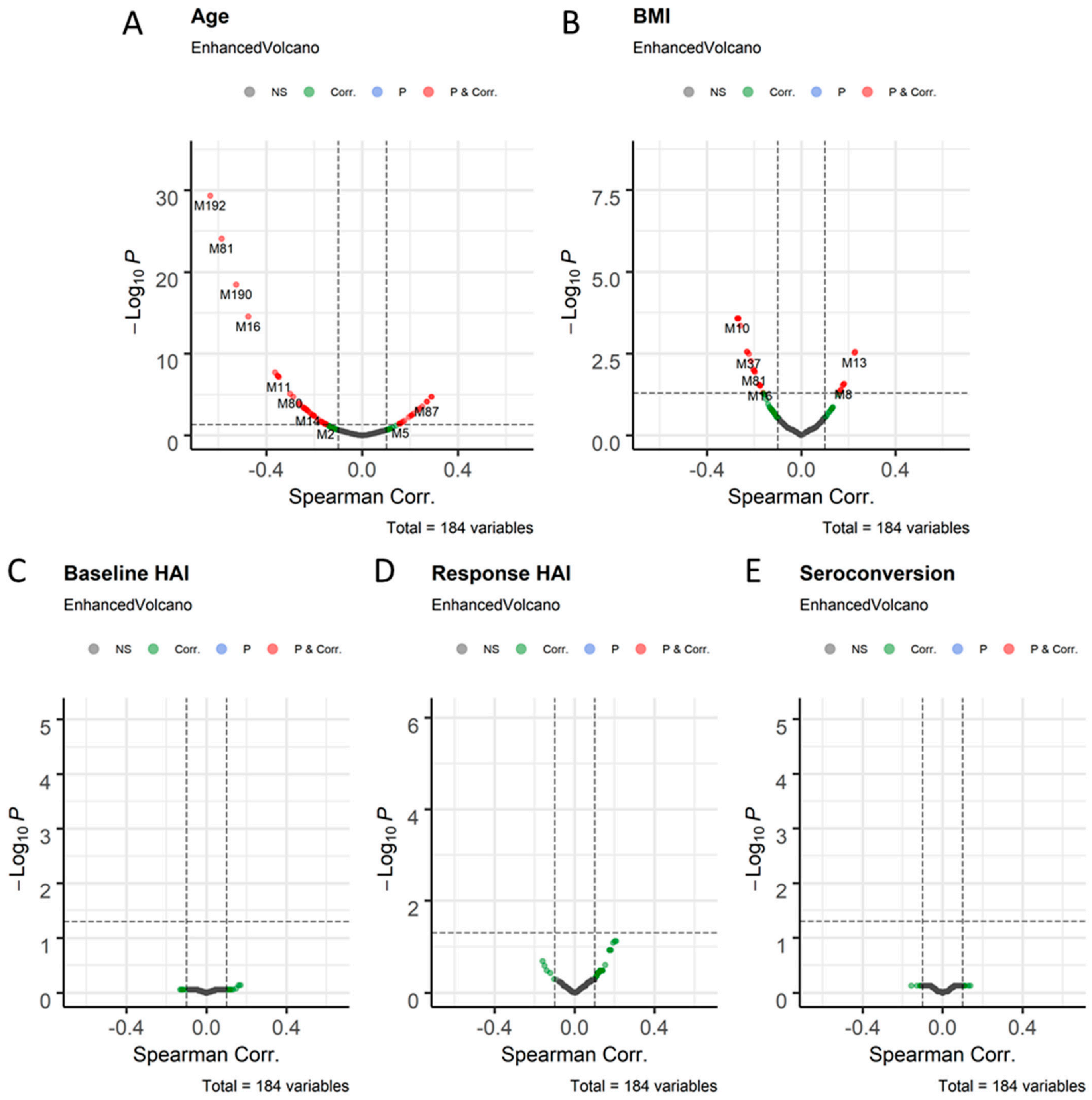

Figure S13. Volcano Plots of Module / Trait relationships. Module/trait relationships are shown between the first principal component of MEGENA modules and selected quantitative traits. *P* on the y-axis refers to FDR (p-values adjusted for multiple testing).

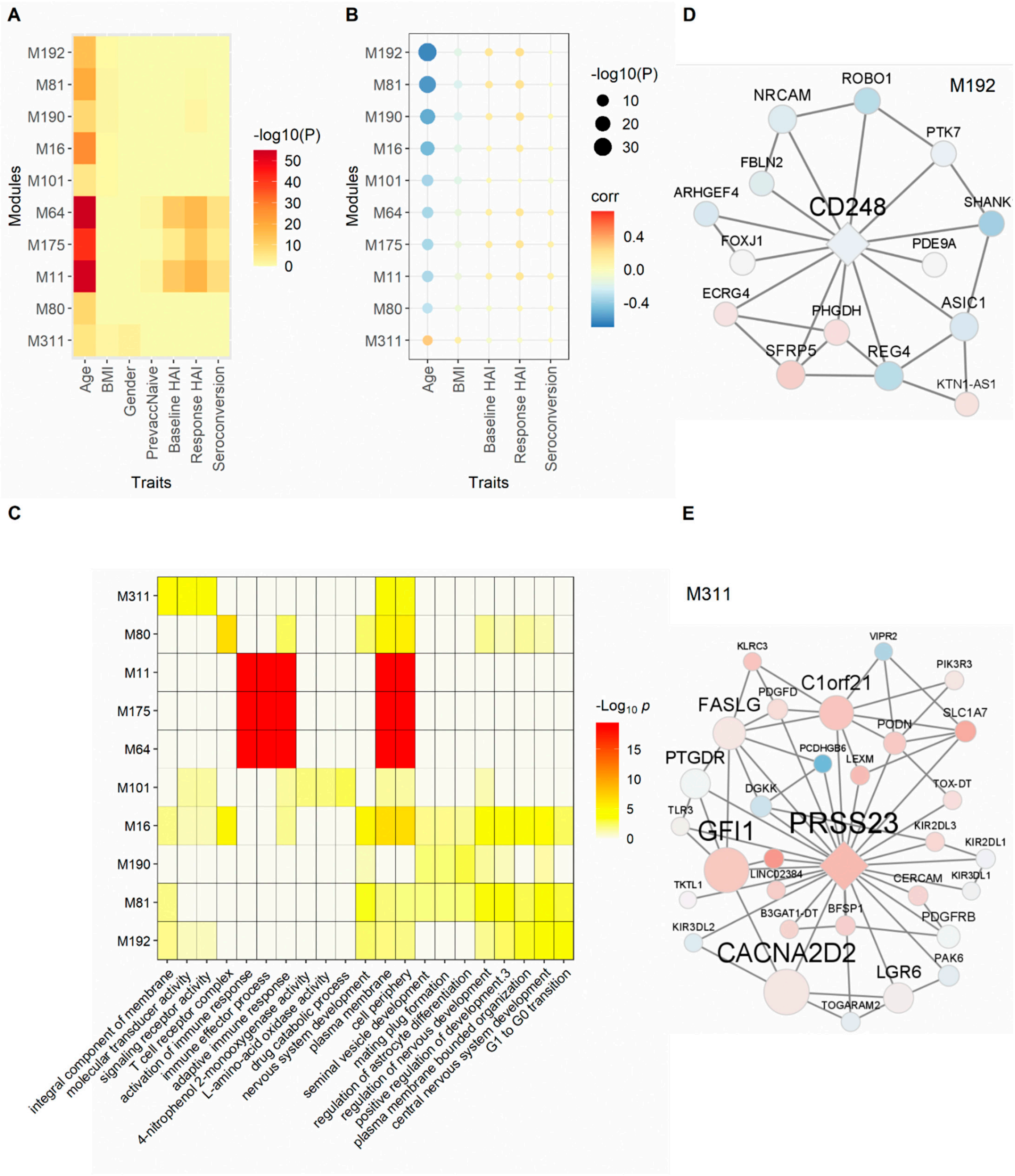

Figure S14. Best ranked modules after significant age response (related to Figure 4). (**A, B**) The 10 best ranked modules are shown using a combined rank based on the correlations related to age. (**A**) The module enrichment for DEGs are depicted in this heatmap. (**B**) The dot-plot shows the correlation coefficients and corresponding P-value of module / trait correlations. (**C**) The functional enrichment of the corresponding modules for GO functions are shown. (**D**) The depicted

module M91 is associated with the elasticity and recoil of the extracellular matrix. (**E**) The vesicle/lysosome component related module M505 is shown. Node colors in (**D, E**) indicate fold change with respect to high versus low average seroconversion, red for increased expression in subjects with high seroconversion compared to subjects with low seroconversion, blue refers to decreased gene expression between these two groups of subjects. The hierarchical relationship of the modules shown in (**A**) and (**B**) are as follows: M11 → M64 → M175; M16 → M80; M16 → M81 → M190/M192; and M17 → M103 → M387 → M706/M707 (see Figure. 4).

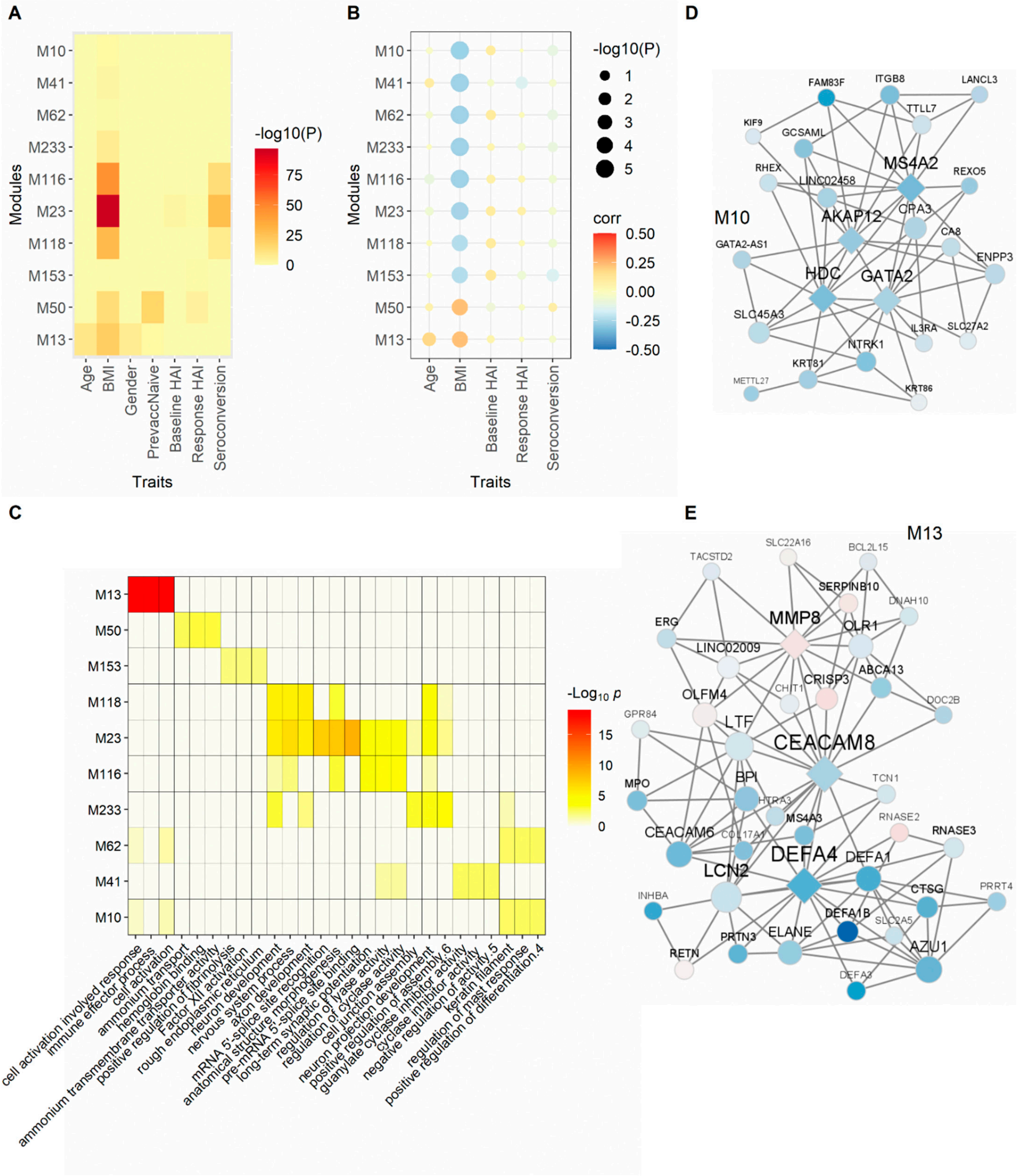

Figure S15. Best ranked modules after significant BMI response (related to Figure 4). (**A, B**) The 10 best ranked modules are shown using a combined rank based on the correlations related to BMI. (**A**) The module enrichment for DEGs are depicted in this heatmap. (**B**) The dot-plot shows the correlation coefficients and corresponding P-value of module / trait correlations. (**C**) The functional enrichment of the corresponding modules for GO functions are shown. (**D**) Module M47 related to cell activation involved in immune response is shown. (**E**) Hemoglobin metabolism related module M8 is depicted. Node colors in (**D, E**) indicate fold change with respect to high versus low average seroconversion, red for increased

expression in subjects with high seroconversion compared to subjects with low seroconversion, blue refers to decreased gene expression between these two groups of subjects. The hierarchical relationship of the modules shown in (**A**) and (**B**) are as follows: M10 → M62; M23 → M116/M118.

.

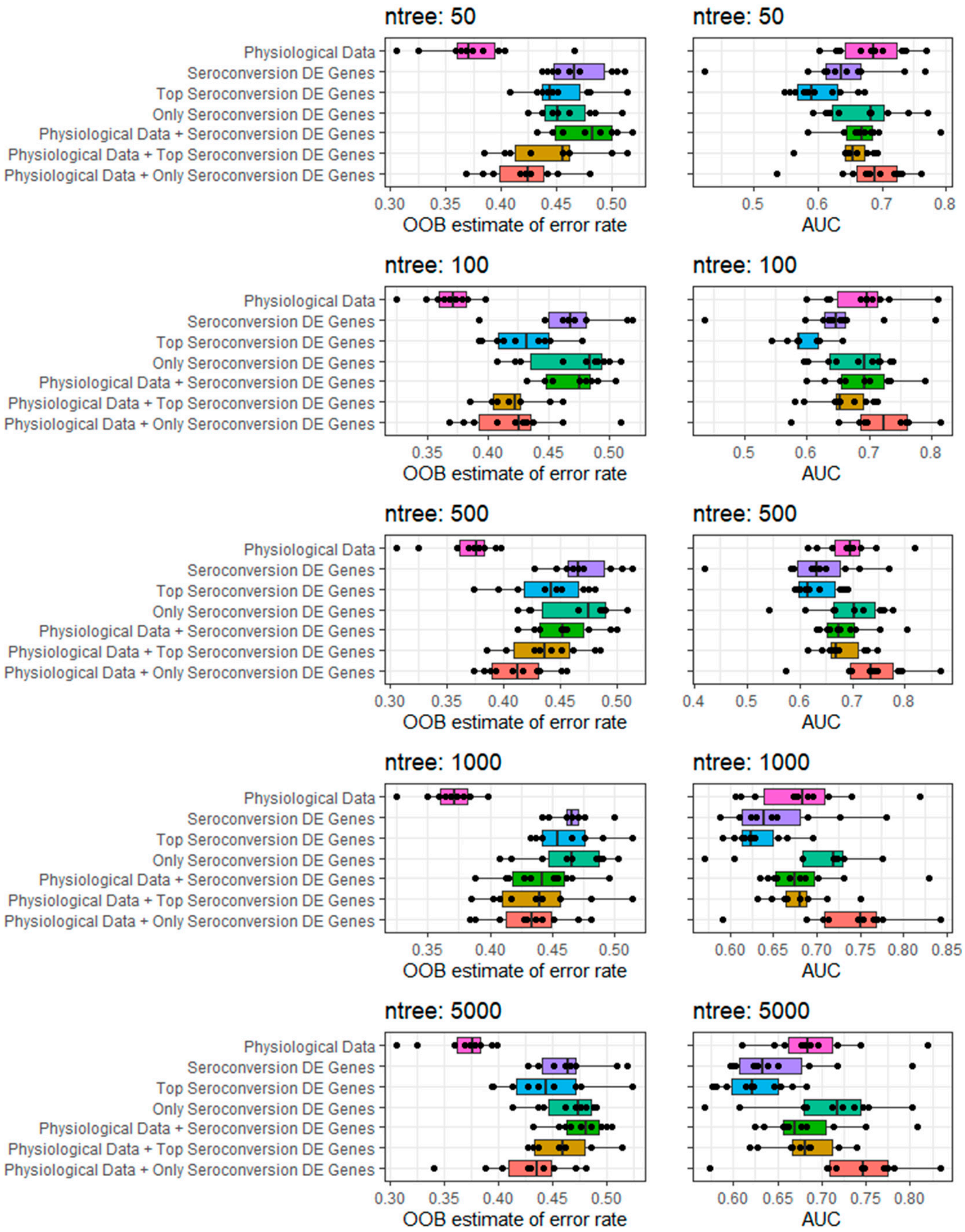

Figure S16. Random forest prediction performance of random forest models varying the ntree parameter. The average out-of-bag (OOB) error rate and average area under the curve (AUC) values were generated using a combination of physiological data and seroconversion DE genes. Each model was generated with 10 iterations with varying ntree values of 50, 100, 500, 1000, and 5000.

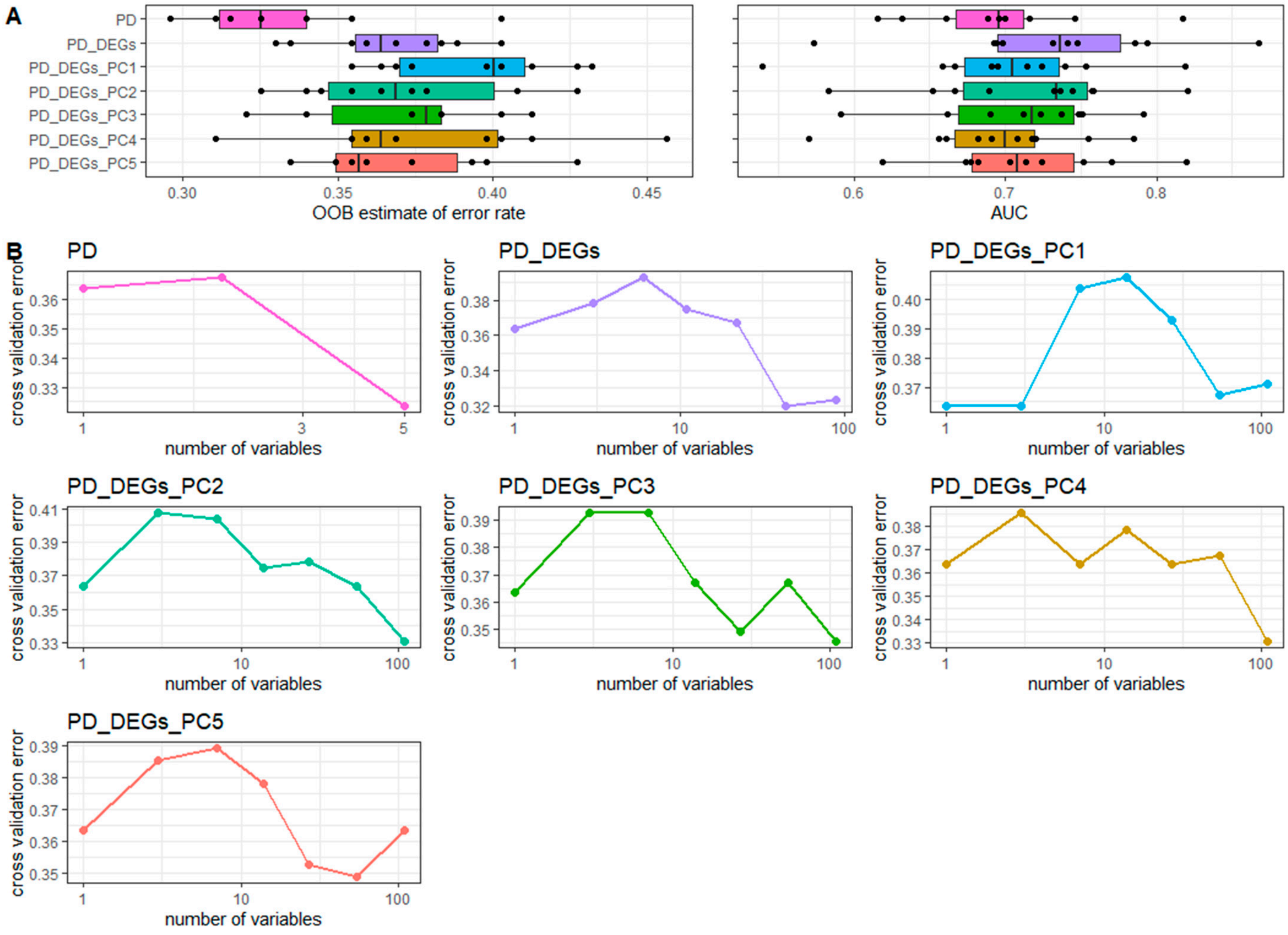

Figure S17. Random forest prediction performance of random forest models generated using clinical, single unit, and aggregated transcriptional data. The average **(A)** out-of-bag (OOB) error rate and **(B)** average area under the curve (AUC) values generated using 10 iterations of random forest models constructed using combinations of physiological data (PD), the 84 differentially expressed genes (DEGs) as a function of seroconversion score, and the principal components 1-5 of the aggregated module data. **(C)** A cross validation analysis plotting a varying number of variables for the clinical data (left), expression data (middle), and both combined (right) random forest models against the resulting cross validation error.

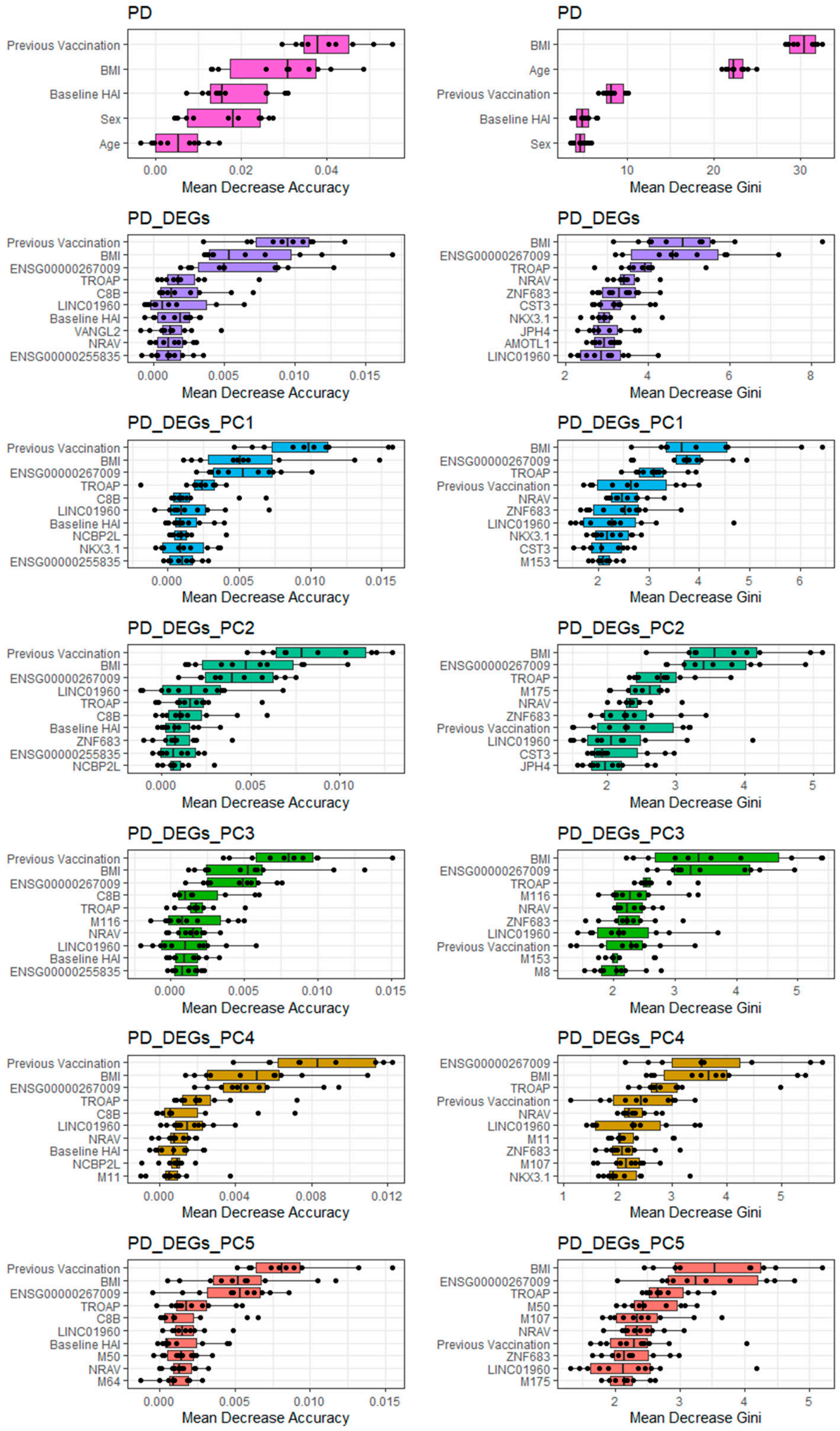

Figure S18. Variable importance values for top 10 clinical data, DEGs, and modules for random forest models generated with clinical data, DEGs, and module principal components. Variable importance measured through mean decrease accuracy and mean decrease in Gini coefficient for random forest modules generated using physiological data (PD), the 84 DEGs as a function of a seroconversion, and one of the five principal components of the expression module dataset.

Supplementary Tables

Table S1. UGA4 study participant information.

Table S2. Gene counts for each study participant obtained from RNAseq performed on whole blood.

Table S3. Univariate differential gene expression analysis of baseline HAI.

Table S4. Univariate differential gene expression analysis of seroconversion.

Table S5. Bivariate differential gene expression analysis of baseline HAI and BMI.

Table S6. Bivariate differential gene expression analysis of seroconversion and BMI.
